# The 5th Annual One Mind Summit: Lessons Learned About “Science Informing Brain Health Policies and Practice”

**DOI:** 10.1089/neu.2016.4821

**Published:** 2017-10-01

**Authors:** Ramona Hicks, Stephen Johnson, Amy C. Porter, Douglas Zatzick

**Affiliations:** ^1^ One Mind, Seattle, Washington.; ^2^ Porter Consulting, Tucson, Arizona.; ^3^ Department of Psychiatry and Behavioral Sciences, University of Washington, Seattle, Washington.

**Keywords:** clinical guidelines, concussion screening, implementation science, T2 translation

## Abstract

Advances in science frequently precede changes in clinical care by several years or even decades. To better understand the path to translation, we invited experts to share their perspectives at the 5th Annual One Mind Summit: “Science Informing Brain Health Policies and Practice,” which was held on May 24–25, 2016, in Crystal City, VA. While the translation of brain research throughout the pipeline—from basic science research to patient care—was discussed, the focus was on the implementation of “best evidence” into patient care. The Summit identified key steps, including the need for professional endorsement and clinical guidelines or policies, acceptance by regulators and payers, dissemination and training for clinicians, patient advocacy, and learning healthcare models. The path to implementation was discussed broadly, as well as in the context of a specific project to implement concussion screening in emergency and urgent care centers throughout the United States.

## Introduction

The concept that scientific evidence should inform clinical practice is widely accepted, but in reality, only about 50% of patients receive recommended evidence-based healthcare. The percentage is even lower (∼25%) for patients with mental health conditions.^1^ To better understand the challenges and opportunities for translating brain research into patient care, we invited experts to share their experiences and perspectives at the 5th Annual One Mind Summit: “Science Informing Brain Health Policies and Practice,” which was held on May 24–25, 2016, in Crystal City, VA.^2^ Prior to the event, participants were given guiding questions related to brain research translation with an emphasis on implementation into clinical practice and policies, and these questions were discussed and refined in subsequent teleconferences. The Summit consisted of a series of panel discussions aimed at examining translational research, both broadly and in the context of a specific project to standardize screening for concussion, also known as mild traumatic brain injury (TBI), in emergency departments (EDs). The highlights and key messages from the Summit discussions are presented in this article.

## Lesson 1: Scientific Discovery and Innovations Follow a Slow and Meandering Path to Clinical Practice

**FIG. 1.**
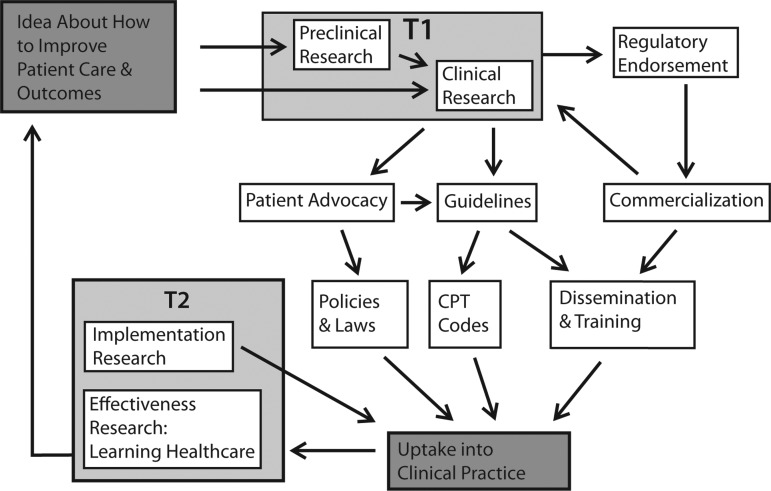
Translating a hypothesis or research question into patient care involves multiple steps, some of which are clustered into phases called T1 and T2. The T1 phase has been referred to as “bench to bedside” and includes the translation of discoveries from preclinical research and smaller human studies into evidence of effectiveness in large-scale clinical trials. The T2 phase, referred to as “bedside to practice” includes research to promote widespread acceptance and implementation of validated diagnostics and effective treatments. T2 research may also include learning health care models and pragmatic clinical trials that incorporate feedback mechanisms to evaluate the patients' responses to treatment, and adapt treatments as needed on an on-going basis. In addition to T1 and T2 research, other key stakeholders and steps may be needed to achieve widespread adoption into clinical care.

The average length of time between scientific discovery and implementation into clinical practice is estimated to be 17 years. ^3–5^ The process is complicated and has been divided into two phases, T1 and T2 (Fig. 1). Often referred to as “bench to bedside,” the T1 phase involves translating basic and pre-clinical discoveries into effective treatments for humans. The second phase, T2, is often referred to as “bedside to practice” and covers the translation of clinical evidence into patient healthcare and policies.^6^ The failure of early discoveries in the T1 phase to demonstrate effectiveness in large human studies has been closely examined over the past few years.^7^ While acknowledging its importance to the entire biomedical research enterprise, the Summit primarily addressed the lesser known issues of the T2 phase.

### Acceptance by professional organizations

The transition from T1 to T2 lacks a clear path, but often involves a review of the evidence and the development of clinical guidelines by professional organizations. “The conscientious, explicit and judicious use of current best evidence in making decisions about the care of individual patients” was championed and formulized in the late 20th century as “evidence-based medicine.”^8^ It is now often referred to as “evidence-based practice” (EBP) to be more inclusive. EBP was so widely embraced by healthcare professionals that there are currently more than 300 governmental and non-governmental agencies grading levels of evidence and creating clinical guidelines.^9^ Not surprisingly, this has led to some confusion, especially when groups tasked with developing consensus guidelines come up with different recommendations. While there are differences, typically basic mechanistic research, pre-clinical studies, and expert opinion are ranked at the bottom, human cohort and case studies in the middle, and randomized controlled trials (RCTs) and meta-analyses at the top.^9^ The evidence is considered along with benefits and risks and the overall burden to patients and providers when developing clinical guidelines.

To harness these independent clinical guidelines activities into better healthcare, the National Academies of Science (NAS) convened experts and published a report, “Clinical Guidelines We Can Trust,” in 2011.^9^ The primary issues identified in the report were: 1) bias in who and what research questions are studied; 2) a lack of diversity and potential conflicts of interest in who ranks and evaluates the evidence; and 3) limited dissemination and training on how to use them for clinicians. To address these concerns, the NAS report recommends greater transparency and diversity of guideline committee members, a separate and independent group to review and grade the evidence, standardization of the process, enhanced dissemination, and more frequent updates.

### Acceptance by regulatory agencies

Early on, there was a rather naïve belief that clinical research alone would result in implementation of prognostic biomarkers to identify the likelihood of a clinical event, disease recurrence, or progression in the ED and other U.S. acute care settings. This has not proved to be the case. For example, numerous publications between 1995–2006 suggested that S100B was a good prognostic biomarker for identifying TBI patients in the ED likely to be computed tomography negative.^10^ This would save patients from radiation exposure, as well as time and expense. Within 4 years in 2010, Scandinavian countries were all using S100B in their EDs as part of their mild TBI guidelines.^11^ In the U.S., however, the Food and Drug Administration (FDA) biomarker qualification process requires much more, including compelling arguments that the biomarker will positively impact clinical care.

The FDA regulated products fall within three broad categories: drugs, biologics, and devices.^12^ The qualification process can be challenging, in part because it continues to evolve and is not aligned across FDA divisions. To assist external stakeholders in understanding the requirements, the FDA has established a Biomarker Qualification Program.^13–16^ A qualified biomarker can be used in any drug development program for the specified context of use (COU) without re-review. The COU is a “comprehensive and clear statement that describes the manner of use, interpretation, and purpose of use of a biomarker in drug development” and is a critical component in determining the level of evidence necessary for qualification. Organizations such as C-Path can also assist researchers with understanding what evidence is needed for acceptance by the FDA, and with coordinating activities among industry, regulatory authorities, government, patient advocacy groups, and academia in the pre-competitive space.^17^

Commercial partners often are needed to provide funding for additional research and validation studies that may be required by the FDA, and later for marketing the innovation and training clinicians. Maintaining funding for these commercial endeavors, given the long interval between initial development and implementation, is a major unmet need.^18^ Although some perceive the FDA as a barrier,^19,20^ it also provides a recognized pathway forward. Many tools and therapies do not require regulatory approval, which in theory should make implementation easier. In some cases this is true, and can result in the proliferation of untested products and unproven treatments (e.g., clinics for stem cell–based interventions).^21^

## Lesson 2: Evidence is Necessary, But Not Sufficient

### Acceptance by payers

Collaborative care, a model that includes primary and specialty care physicians, a care coordinator, and health informatics to ensure continuity, was started 20 years ago, and there have been hundreds of clinical trials to evaluate its effectiveness.^22,23^ Indeed, there were more than 74 randomized clinical trials on depression alone that demonstrated improvements in short- and long-term outcomes, fewer suicides, and increased work productivity—all without increases in cost.^24^ Despite this vast body of evidence, collaborative care is still not standard practice because of a lack of reimbursement by payers for physician-to-physician contact. However, because of newly approved Current Procedural Terminology (CPT) codes for reimbursement by the Centers for Medicare and Medicaid Services, collaborative care may soon be a reality. The CPT codes are a common requirement for reimbursement by other payers, too.

Although payers rely heavily on safety and clinical effectiveness research, they also must consider costs and comparative effectiveness. Comparative effectiveness research has been difficult to fund in the past, but new opportunities now exist at the Patient Centered Outcomes Research Institute (PCORI).^25^ Also, the National Institutes of Health (NIH) recently created an Implementation Science Division to support “the scientific study of methods to promote the systematic uptake of proven clinical treatments, practices, organizational, and management interventions into routine practice, and hence to improve health. In this context, it includes the study of influences on patient, healthcare professional, and organizational behavior in either healthcare or population settings.”^26,27^

Segmentation of the healthcare system presents another challenge to gaining acceptance by payers because even if a treatment saves money to the “system” overall, it may cost more in one segment. For example, longer inpatient rehabilitation stays may reduce the number of readmissions to the hospital after discharge, but whoever pays for one segment may not be rewarded for saving money in another segment. Also, CPT codes that are needed for reimbursements by insurance companies and other providers are becoming even more difficult to obtain for new devices and treatments because of greater use of “bundled” payments. Improvements in health informatics that make it easier to measure and hold people accountable for outcomes are needed to facilitate changes in reimbursement policies.

### Patient advocacy

Patients, caregivers, and the public may be the most powerful forces in moving scientific advances into patient care. Even the extraordinary life-saving polio vaccine and immunodeficiency virus (HIV) treatments needed assistance from patients, caregivers, and the public to mobilize the steps needed for rapid widespread implementation.^28,29^ While the benefits of patient advocacy can be enormous, there also are risks. Commercial vendors and unregulated industries commonly target patients and caregivers with “direct-to-consumer” advertising to use their products and/or advocate for their treatments, despite limited evidence or lack or regulatory approval in some cases.^21^

To address the benefits and risks, greater inclusion of patients and caregivers in the research process is critical. However, patient and caregiver participation usually depends on volunteerism, which can be a significant barrier for people with limited resources. Patient advocacy organizations and online platforms, such as PatientsLikeMe,^30^ can be helpful in balancing patient perspectives and scientific evidence. The recent attention by regulatory agencies on “Patient Focused Drug Development” and the role of advocacy organizations in driving regulatory advances is timely.^31,32^ Similarly, the PCORI aims to include patients in the research design process.^25^

### Legislation

Patient advocates often are instrumental in enacting legislation to effect and sustain changes in practice. An example is the Zackery Lystedt Law, a comprehensive youth sports concussion safety law, which was enacted in Washington State in 2009. Zackery Lystedt was 13 years old when he sustained a catastrophic brain injury playing football. To prevent this from happening to other children, a statewide concussion education campaign for coaches and school administrators was started and enthusiastically embraced. However, when those individuals left schools, the knowledge often left with them, and the programs were not consistently sustainable. Therefore, a broad-based coalition was built to lobby for a legislative solution. The legislation included three tenets: 1) education for athletes, parents, and coaches; 2) removal from practice or play at the time of a suspected concussion; and 3) clearance from a licensed healthcare professional knowledgeable in the evaluation and management of concussion prior to return to play.^33^ Despite its own challenges with implementation, the law has increased reporting of concussion, improved coaches' knowledge about concussions, and resulted in more medical care provided for this injury.^34,35^ A missing piece is a lack of standardized screening tools and management guidelines in EDs to ensure that patients who seek care are evaluated and given consistent discharge information based on the best available evidence. This would close the loop and transform the increased awareness and knowledge about sports concussions into consistent actions to help keep children safe.

While legislation can be a powerful tool for bringing scientific advances to patient care, another example illustrates the need for using it judiciously as it can be a barrier to scientific advances. The Rory Staunton Regulation, named for a teenager who died of sepsis after being sent home from the ED, is legislation that also was intended to increase compliance with best practices. This law requires EDs in New York to screen for and recognize sepsis, even though the mandated screen is now outdated because the criteria for diagnosing sepsis have been changed.^36^ Therefore, implementation of any laws that require screening for a disease that may not yet be well understood require careful and adaptable guidelines. In addition, patient advocacy groups and others with special interests must recognize that they are competing for finite resources and time within healthcare systems.

## Lesson 3: It Ends at the Beginning

### Learning healthcare models

Recent commentary has expanded the T2 pathway to include feedback loops that inform both individual and population level care.^37,38^ Kaiser Permanente, a health maintenance organization, is developing and testing an innovative approach called “Feedback Informed Care.”^39^ Rather than relying on the passive diffusion of discovery into health plans, they are surveying patients and collecting data on patient reported outcomes in real-time to target and drive adjustments in treatments. “Feedback Informed Care” is especially needed in mental health where data have not been routinely collected and patient acceptance is uncertain. The strategy is “screen, measure, intervene—repeat” to achieve better outcomes.

Several key findings have emerged from “Feedback Informed Care.” Data show that patients with mental health conditions such as depression who stay in treatment do get better and that concentrated care at the beginning produces a better outcome later (Donald Mordecai, personal communication, May 25, 2016). It also has been shown that individual care sessions and a treatment alliance/collaborative care process that follows the patient is critical to treatment of mental health. These changes in practice are likely to require changes in staffing and other resources, which will happen only when compelling evidence is available. Long-term patient outcome data and longitudinal studies, which are difficult to fund and carry out, also are needed to inform learning healthcare models.

“Pragmatic trials”—trials that focus on targets for change and decisions about practice—are another way to more rapidly implement advances in diagnostic tools and treatments.^40^ The “Trauma Survivors, Outcomes and Support” (TSOS) study, funded as part of the NIH Healthcare System's Research Collaboratory, is using a pragmatic trial to evaluate a screening tool and intervention for post-traumatic stress disorder (PTSD) and other co-morbid disorders.^41^ The lack of criteria for diagnosing PTSD and the limited understanding of the link between it and other co-morbid conditions are major barriers to developing effective treatments. TSOS uses a hybrid effectiveness–implementation model to simultaneously provide an evidence base for screening and intervention. Timely implementation of research findings is a key aspect of the model, with the clear understanding that the treatment guidelines and policies may require some changes as more information is collected over time. In addition to the innovative approach, this study also highlights the importance of partnering with professional societies. TSOS is actively collaborating with the American College of Surgeons (ACS) to facilitate the use of the pragmatic trial results and ensure that important findings are translated into action and improved healthcare practice and policy, and not just into publications.

The TSOS process starts with a 10-domain PTSD risk screen that is embedded into electronic medical records (eMRs),^42^ followed by a brief, trauma center–based stepped collaborative care intervention for patients who screen positive. Step One, the initial intervention, is a modified collaborative care model with one case manager and the establishment of a continuous, healing relationship with the patient over 6 months after the acute care injury admission. Step Two builds in further assessments of PTSD, suicide, drug and alcohol use, sleep, and medications, if necessary. Continued symptoms require yet another level of care and intervention in order to keep the patient engaged and improve outcomes that can include referral to specialty clinics. Importantly, the initial interventions are enacted in the trauma center or ED, because patients frequently do not follow up on referrals.^42–44^

Pragmatic trials that use an implementation science model hypothesize that interventions will get better by collecting data and acting on them in real time. With PTSD, TBI, and other brain-health disorders, there are lots of variables and interventions do not necessarily distill down to one or two options. The TSOS trial makes use of a stepped-care randomized design, which targets different components with an evidence base behind each intervention. The study tracks individual patient responses to interventions toward the goal of personalized medicine. For example, in subgroup analyses of a large multi-center trial of alcohol screening and brief intervention, the study team discovered that an intervention in the acute care setting that decreased drinking in at-risk patients was not effective in at-risk patients with TBI.^45^ This example also highlights the need for understanding brain disorders in the context of co-morbid conditions in order to provide effective treatments.

## Lesson 4: Implementing Small Incremental Advances May Be the Hardest of All

In 2009, the Army and Marines worked with the medical community to design and deploy a protocol in Iraq and Afghanistan to prevent military personnel from sustaining a second impact syndrome. A civilian blue-ribbon panel of experts put together a protocol in 36 h (General Peter Chiarelli [Ret.]), personal communication, May 25, 2016). Within 6 weeks, anyone in two combat theaters who was in a vehicle damaged by an explosion, was within 50 m of an explosion, lost consciousness, was in a structure when an explosion went off, or was command directed was immediately screened for concussion. Those screening positive also were restricted from further engagements, screened again at 24 h, and sent to a concussion recovery center, if necessary. This protocol was later adopted by the U.S. Department of Defense.^46^ Similar screening protocols have been validated and are widely used in the assessment of mild TBI in professional sports.^47,48^ However, there is no “gold standard” for concussion screening in civilian EDs and urgent care centers.^49^

There are roughly 2.5 million documented visits/year in EDs for TBI,^50,51^ and potentially 1.0–1.5 million more based on reports of missed cases.^52^ Patient screening and education is important because more than 85% of patients with TBI are discharged home from EDs.^50^ A significant number (∼ 25%) will develop persistent symptoms lasting up to 1 year after injury^53^ or may be at greater risk for a second concussion.^54^ Several professional organizations have reviewed the evidence on sports concussion and have all recommended: a) discontinuing play on Day 1; b) clearance by a healthcare provider; and c) gradual stepwise return to physical activities before return to play.^55^ Similar guidelines have also been recommended for non-sports–related concussion.^56^ With this information in hand, leveraging the military and sports protocols to implement a standardized concussion screening protocol into EDs seemed relatively straightforward.

However, this has not been the case for “Screen, Inform, Prevent,” a grassroots effort launched in late 2014 to standardize concussion screening and management in EDs and urgent care centers throughout the U.S.^57^ The objectives are to: 1) reduce the number of missed cases of mild TBI, a problem reported even for Level 1 civilian trauma centers^52^; 2) provide evidence-informed discharge guidelines for children and adults seeking acute trauma care in EDs and other health care settings; and 3) create a cost-effective national registry. The proposed screening tool for EDs is basically a shorter, simpler, electronic version of the evidence-based military and sports screens.^57^ It starts with two triage questions to determine if there was an incident of trauma to the head and alterations in consciousness. If the answers to both questions are positive, it triggers an alert to the physician to further assess the injury characteristics, the patient history, symptoms, and physical findings relevant to concussion. For patients who screen positive, the plan is to inform them of the diagnosis and also provide basic management guidance via written standardized discharge instructions developed by the Centers for Disease Control and Prevention (CDC).^58^

Widespread adoption of a concussion screening tool needs to address and overcome the very real and unique barriers faced in high-paced ED environments. EDs are not only frontline providers for life-threatening emergencies, but also for a wide range of medical conditions. In this role, they often are expected (or mandated) to use several other screening tools, such as screening for alcohol misuse, domestic violence, HIV, sepsis, and suicide risk, car seat use, tobacco use, hypertension, vaccination histories, and fall risks.^59^ To justify adding another screening tool, there must be compelling evidence that it will inform and significantly improve patient care. Currently, the lack of pharmacological and other therapies for TBI has led to the perception that nothing can be done. In reality, patient education and discharge instructions on what to expect and how to manage symptoms have been shown to improve outcomes.^60–65^

The CDC and American College of Emergency Physicians (ACEP) have developed guidelines for concussion management in EDs, but they do not include a specific protocol for screening.^49,56^ Acceptance and endorsement of a standardized screening tool by the ACEP and other organizations, such as the Brain Trauma Foundation and the ACS, are critical next steps. The ACS in particular has had notable success at implementing consensus-driven, evidence-informed guidelines because they require them as part of their process for accreditation.^66^ The ACS process is framed around accountability for patient outcomes and involves a four-part model: 1) the development of standards; 2) the development of infrastructure to implement the standards; 3) the development of databases to assess the performance of the standards in practice; and 4) use of external peer review to verify performance and assure the public that the standards are being followed.

### Creating a learning healthcare model for concussion

Another reason to implement a standardized concussion screening tool that is incorporated into eMRs is the potential to create a patient registry and collect data about the actual numbers, characteristics, and management of mild TBI patients seen in EDs. Pediatricians also could benefit from access to this tool and could further contribute to the registry because they provide most of the follow-up care for children with concussions.^67,68^ The value added for patients is the opportunity to collect better data (not necessarily more data) that can be used to evaluate and monitor alterations in function and recovery after concussion, and apply this information into a learning health care system. A grassroots approach has been successfully employed in the past to share data and advance care for inflammatory bowel syndrome.^69^

In collaboration with EPIC, a major heathcare software company, the concussion screening tool has been incorporated into eMRs at one site. This was not trivial, and required early collaboration of clinician–scientists, information technology and analytics experts to ensure close attention to the clinical workflow. The screening tool at this site is currently being beta tested for feasibility in an ED to evaluate the time required, workflow and to identify other issues. The results will inform future iterations of the EPIC tool, as well as tools built for other eMR platforms. However, because there is no standard, solutions will need to be customized to account for variations in eMR platforms, for differences in workflow among pediatric and adult EDs and urgent care centers.^70^

## Summary and Recommendations

Numerous factors contribute to the slow and limited uptake of scientific discoveries into clinical care. The relatively new field of implementation science has emerged to study these factors and develop effective strategies for overcoming them. Implementation science alone, however, is unlikely to resolve all of the problems because changing clinical practice requires actions by stakeholders outside of the research realm. Therefore, researchers are advised to “take a crude look at the whole,” (a term coined by Nobel laureate Murray Gell-Mann for addressing complex problems), and roughly map out the steps and stakeholders needed to move their discoveries into patient care. This will set the stage for early collaborations with the larger stakeholder community, and ideally accelerate the transitions from one phase to the next. Ultimately, this should lead to more science-informed brain health policies and practice, a goal that is not only worthy but also imperative.

Pragmatism asks its usual question. “Grant an idea or belief to be true,” it says, “what concrete difference will its being true make in anyone's actual life?”

– *William James*
